# Identifying the changing age distribution of opioid-related mortality with high-frequency data

**DOI:** 10.1371/journal.pone.0265509

**Published:** 2022-04-20

**Authors:** Lauren A. Paul, Ye Li, Pamela Leece, Tara Gomes, Ahmed M. Bayoumi, Jeremy Herring, Regan Murray, Patrick Brown

**Affiliations:** 1 Health Protection, Public Health Ontario, Toronto, Ontario, Canada; 2 Dalla Lana School of Public Health, University of Toronto, Toronto, Ontario, Canada; 3 Knowledge Services, Public Health Ontario, Toronto, Ontario, Canada; 4 Health Promotion, Chronic Disease and Injury Prevention, Public Health Ontario, Toronto, Ontario, Canada; 5 Substance Use Service, Women’s College Hospital, Toronto, Ontario, Canada; 6 Department of Family and Community Medicine, University of Toronto, Toronto, Ontario, Canada; 7 Li Ka Shing Knowledge Institute, St. Michael’s Hospital, Toronto, Ontario, Canada; 8 ICES, Toronto, Ontario, Canada; 9 Leslie Dan Faculty of Pharmacy, University of Toronto, Toronto, Ontario, Canada; 10 Institute for Health Policy, Management and Evaluation, University of Toronto, Toronto, Ontario, Canada; 11 MAP Centre for Urban Health Solutions, St. Michael’s Hospital, Toronto, Ontario, Canada; 12 Department of Medicine, University of Toronto, Toronto, Ontario, Canada; 13 Office of the Chief Coroner for Ontario, Toronto, Ontario, Canada; 14 Public Health Agency of Canada, Ottawa, Ontario, Canada; 15 Department of Statistical Sciences, University of Toronto, Toronto, Ontario, Canada; Universita degli Studi di Milano-Bicocca, ITALY

## Abstract

**Background:**

Opioid-related mortality continues to rise across North America, and mortality rates have been further exacerbated by the COVID-19 pandemic. This study sought to provide an updated picture of trends of opioid-related mortality for Ontario, Canada between January 2003 and December 2020, in relation to age and sex.

**Methods:**

Using mortality data from the Office of the Chief Coroner for Ontario, we applied Bayesian Poisson regression to model age/sex mortality per 100,000 person-years, including random walks to flexibly capture age and time effects. Models were also used to explore how trends might continue into 2022, considering both pre- and post-COVID-19 courses.

**Results:**

From 2003 to 2020, there were 11,633 opioid-related deaths in Ontario. A shift in the age distribution of mortality was observed, with the greatest mortality rates now among younger individuals. In 2003, mortality rates reached maximums at 5.5 deaths per 100,000 person-years (95% credible interval: 4.0–7.6) for males around age 44 and 2.2 deaths per 100,000 person-years (95% CI: 1.5–3.2) for females around age 51. As of 2020, rates have reached maximums at 67.2 deaths per 100,000 person-years (95% CI: 55.3–81.5) for males around age 35 and 16.8 deaths per 100,000 person-years (95% CI: 12.8–22.0) for females around age 37. Our models estimate that opioid-related mortality among the younger population will continue to grow, and that current conditions could lead to male mortality rates that are more than quadruple those of pre-pandemic estimations.

**Conclusions:**

This analysis may inform a refocusing of public health strategy for reducing rising rates of opioid-related mortality, including effectively reaching both older and younger males, as well as young females, with health and social supports such as treatment and harm reduction measures.

## Introduction

Opioid-related mortality remains a crucial public health issue in North America, which has been further exacerbated by the ongoing COVID-19 pandemic. In the United States, the number of opioid-related deaths nearly quadrupled between 2003 and 2019 [1, 2], and in 2016 opioids were named the fifteenth highest cause of years of life lost [3]. In Canada, opioid-related mortality increased from 7.8 deaths per 100,000 persons to 16.7 deaths per 100,000 persons between 2016 and 2020, and had already reached a rate of 19.6 deaths per 100,000 persons in the first quarter (January to March) of 2021 [4]. Ontario, the most populous province in Canada, has seen a particularly large increase in opioid-related mortality over the last two decades; mortality rates jumped more than fivefold between 2003 and 2020 [5]. Of particular note, there was a 79% increase in the number of opioid-related deaths between February 2020 (the month prior to Ontario’s state of emergency declaration) and December 2020 [6].

Large increases in morbidity and mortality involving opioid drugs were initially attributed to over-prescribing of opioids for pain [7]. As prescribing rates slowed, following widespread recognition of the risks of opioid analgesics and close monitoring/regulation of prescribing [8–10], several regions observed some stabilization or decline in deaths from prescription opioids [11–16]. However, due to the emergence of highly potent non-pharmaceutical opioid drugs such as fentanyl and fentanyl analogs [7], deaths from synthetic opioids have continued to rise [11–13, 16]. Furthermore, recent pandemic restrictions and physical distancing requirements have created high risk environments for opioid overdose. Barriers to accessing treatment and harm reduction services, disrupted drug supply, and negative impacts on mental health are all thought to have contributed to new spikes in mortality [6, 17, 18]. These events have collectively led to significant increases in opioid-related deaths over time [6, 11–14, 16, 18], as well as shifts in the demographic makeup of deaths [12, 19–21]. Specifically, some regions have observed large increases in opioid-related mortality rates for younger individuals, surpassing older individuals [12, 20, 21], as well as differences in trends for males versus females [11, 12, 14, 16, 20, 22–24]. Therefore, it is of interest to better quantify and understand how opioid-related mortality distributions have changed over the last two decades by age and sex.

This study sought to provide an updated picture of how opioid-related mortality has changed in Ontario between 2003 and 2020, in relation to age and sex, through an application of novel Bayesian modelling. Previous studies have been limited by modelling age in groups; however, binning data into age groups leads to the creation of artificial age breaks, to which the results can be sensitive, especially when mortality distribution by age changes over time. In addition, age groups can mask important trends and features in the data, such as peaks or troughs in mortality rates for particular ages. Our Bayesian modelling approach is able to utilize continuous age data that are sparse, allowing flexible patterns for age-time interaction by sex. We also used the model to explore how mortality trends might continue into 2022, considering both pre-COVID-19 and post-COVID-19 courses, to provide 20-year pictures of the opioid mortality crisis in Ontario. These results may provide insight for tailored public health interventions to more effectively reduce rising rates of opioid-related mortality in Ontario or other demographically similar regions.

## Methods

### Study population

Data on opioid-related mortality in Ontario between January 2003 and December 2020 was obtained from the Office of the Chief Coroner. Following the Ontario Coroners Act, all deaths from non-natural causes are investigated by physicians at the Office of the Chief Coroner. The cause and manner of death are determined by the death investigation and a post-mortem examination by a pathologist, informed by interpretation of toxicology testing [6, 21, 25]. Opioid-related deaths are defined as deaths where acute drug toxicity involving opioids, including opioids of both pharmaceutical and non-pharmaceutical origin, is considered as directly contributing to the cause of death based on the text in the cause of death field (noting standardized ICD coding is not used by the Office of the Chief Coroner) [6, 21, 25]. The definition excludes deaths due to chronic substance use, medical assistance in dying, trauma where an intoxicant contributed to the circumstances of the injury, and deaths classified as homicide [25]. Additionally, the Office of the Chief Coroner collects demographic information on each individual’s age and sex, as well as information on date of death and whether the death was deemed to be accidental [6]. Accidental manner of death is defined as instances when the death resulted from an injury event where death was not intended, foreseen, or expected; inflicted injury did not cause or substantially contribute to the death [25]. Apart from accidental, other manners of death include natural deaths (i.e., due to natural disease or a known complication), suicide, and deaths where the manner cannot be clearly identified (undetermined manner) [25].

Only accidental opioid-related deaths were included in the cohort as they represented the vast majority of deaths, and interventions to reduce mortality may differ for accidental versus other manners of death [25]. We further restricted to individuals that had complete demographic information (77 individuals excluded), as well as individuals between the ages of 15 and 69 years as opioid-related deaths were rare outside of this age range (96 individuals equating approximately 0.8% of deaths after excluding individuals with missing demographic information), and it becomes challenging to assign a single cause of death to older individuals. To calculate rates, we used Statistics Canada’s annual population estimates for the province of Ontario broken down by age and sex [26].

Use of these data was approved in writing by the research ethics board at Public Health Ontario (ID: 2017–065.01). The de-identified data were transferred to Public Health Ontario in SAS dataset format (.sas7bdat) through a secured Microsoft SharePoint site that only select project members had access to. The analysis was done using R v.4.11 and results were written back out to the secure SharePoint site.

### Statistical analysis

We applied Bayesian mixed-effects Poisson regression to model opioid-related mortality over time and by age, with person-years (derived from population estimates) accounted for as an offset in the model. The time trend was modelled as changing monthly (i.e., January 2003 –December 2020), and the age effect varying by single-year age groups (i.e., 15–69), with random walk models encouraging the time trend and age effect to vary smoothly. Although monthly mortality counts were often zero for some ages, the random walk models borrow strength across nearby ages and time points to produce smooth risk estimates when counts are low. The interaction of age and time was modelled as a two-dimensional random walk, which allowed mortality rates to change more quickly for some ages than others in a smooth manner (e.g., the time trend for age 50 should be similar to the time trend for age 49). The smoothness or roughness of the age and time effects, as well as their interaction, is governed by the variance parameters, which were estimated from the data (S1 Appendix). If the observed mortality counts changed quickly in irregular ways (e.g., with ages 49 and 50 being unrelated), the estimated variance parameters would be large and trends identified would be rough. Bayesian prior distributions on the variance parameters were chosen to encourage smoothness, with exponential priors on the standard deviations (S1 Appendix). Seasonal variation was modelled with indicator variables for calendar month (i.e., January—December) that were constrained to sum to zero for easy interpretation. Additionally, an independent random effect for time in months (i.e., January 2003 –December 2020) allowed for over-dispersion, or mortality counts exhibiting more variation than the Poisson distribution can accommodate. The model was stratified on sex; males and females had distinct time trends, age effects, and seasonal effects, although variance parameters were shared. As this Bayesian inference was implemented with Integrated Nested Laplace Approximation (INLA), the estimated rates were the 50 percentile (median) of the posterior distribution, and the credible intervals were the 2.5 and 97.5 percentiles of the posterior distribution, which were obtained directly from the posterior probability density function. Further details on the model and prior specifications, including plots of monthly effects (S1a and S1b Fig) and priors and posterior distributions for the standard deviations of the random effects (S2a–S2d Fig), can be found in the S1 Appendix.

To visualize the results, estimated rates were plotted by age over time for each sex using 2D heat maps as an overview. In order to better understand how mortality changed over time, estimated mortality distributions over age were plotted for selected years. We further plotted relative risk over time for selected ages to understand how the rate of increase in mortality differed by age for each sex, and this information was also summarized using 2D interaction heat maps.

We then used the model to explore mortality trends up to December 31, 2022 in order to examine how opioid-related mortality might change over the next 2 years. This exploration was done assuming trends will continue without disturbance, meaning that all parameters estimated from the model using the observed data would remain constant except for time. To assess the impact of the COVID-19 pandemic on mortality rates, we re-fit the model to pre-pandemic data only (January 2003 to February 2020), and used the model to explore mortality up to December 31, 2022 again as comparison.

## Results

During the 18-year study period, there were a total of 11,633 opioid-related deaths that met our inclusion criteria, and 72% were male (Table 1). Accidental deaths accounted for 82% of total deaths. For both sexes, deaths were concentrated among the 25 to 54-year age group; the mean age for males was 41 years and the mean age for females was 42 years. In comparison, the source population (the entire population of Ontario aged 15 to 69) was estimated by Statistics Canada to be 10,758,617 persons in 2020, and 50% male [26]. The mean age for both males and females was 42 years. Population estimates for 2003 and 2020 by age group and sex can be found in S1 Table.

**Table 1:** Characteristics of opioid-related deaths in Ontario (January 1, 2003 to December 31, 2020).

	Male (N = 8,429)	Female (N = 3,204)
**Age group (N, %)**		
15–24 years	721 (8.6)	265 (8.3)
25–34 years	2,237 (26.5)	692 (21.6)
35–44 years	2,245 (26.6)	777 (24.3)
45–54 years	2,030 (24.1)	913 (28.5)
55–64 years	1,073 (12.7)	493 (15.4)
65–69 years	123 (1.5)	64 (2.0)
**Two-year periods (N, %)**		
2003–2004	286 (3.4)	104 (3.2)
2005–2006	388 (4.6)	157 (4.9)
2007–2008	438 (5.2)	190 (5.9)
2009–2010	534 (6.3)	241 (7.5)
2011–2012	596 (7.1)	277 (8.6)
2013–2014	725 (8.6)	304 (9.5)
2015–2016	920 (10.9)	395 (12.3)
2017–2018	1,796 (21.3)	627 (19.6)
2019–2020	2,746 (32.6)	909 (28.4)
**Quarter (N, %)**		
January–March	1,902 (22.6)	725 (22.6)
April–June	2,203 (26.1)	794 (24.8)
July–September	2,111 (25.0)	792 (24.7)
October–December	2,213 (26.3)	893 (27.9)

### Model fit

We plotted modelled mortality rates against observed mortality rates in order to visualize the model fit. In the face of sparse data, we plotted the model estimates for a given age and sex overlaid with observed mortality rates for the same sex, but instead for the 5-year age group surrounding the given age and at a lower temporal resolution by binning the time at 3-month intervals. An example of the model fit for males aged 35 (observed mortality rates for males aged 33–37) can be found in S3 Fig. Plotting other ages and females yielded similar alignment between the model estimates and the raw rates.

### Historical mortality trends

**Fig 1:**
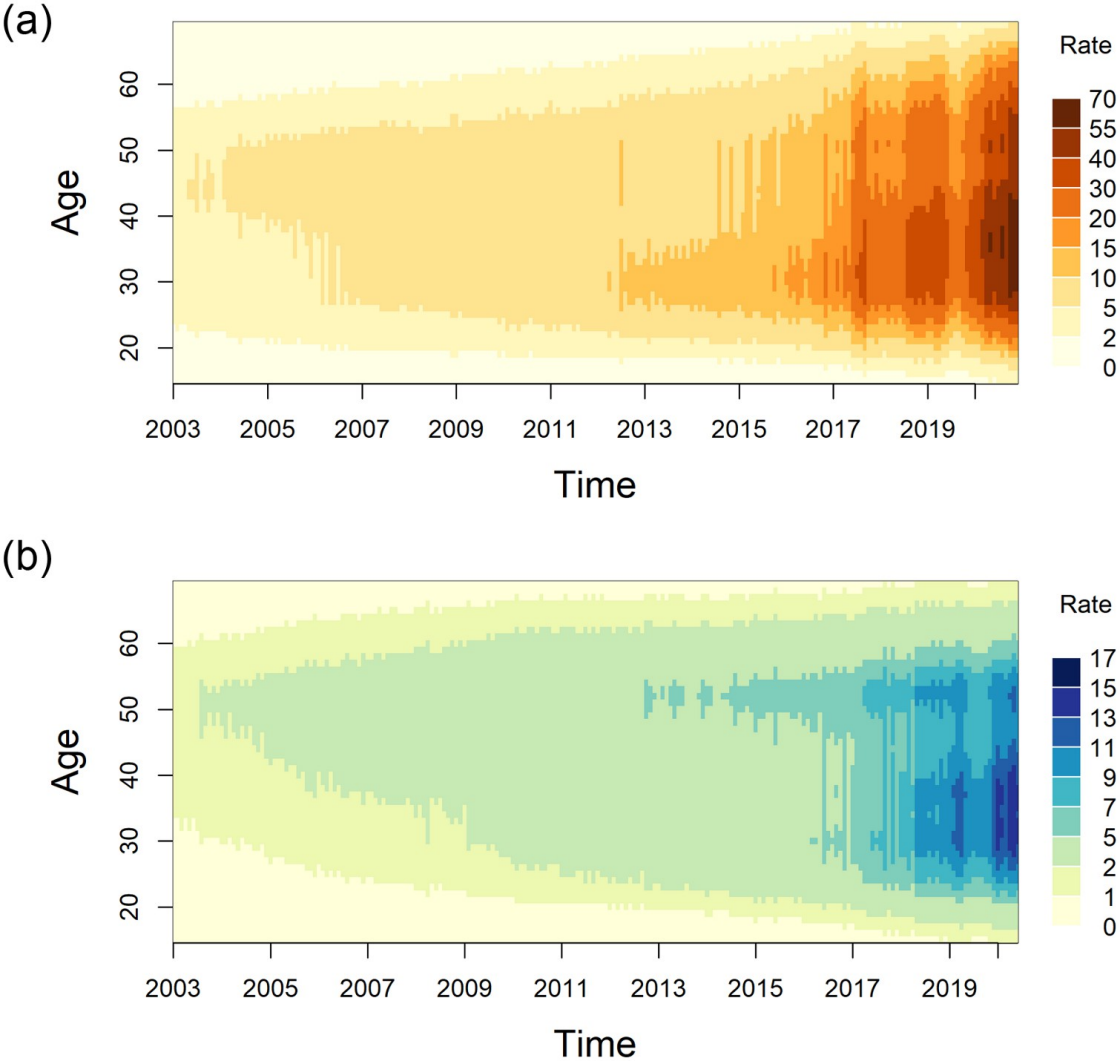
Opioid-related mortality rate per 100,000 person-years, by age and date of death in months (January 2003 to December 2020). (a) Males. (b) Females.

Opioid-related mortality rates have increased substantially for both sexes and across all ages over time, shown by the colour gradation in Fig 1a and 1b, which present heat maps of monthly modelled rates by age (corresponding to the term *exp*[*X*_*t*_*β*_*i*_ + *U*_*i*_(*a*) + *V*_*i*_(*t*) + *W*_*i*_(*a*, *k*) + *Z*_*it*_] in the S1 Appendix). In 2003, mortality rates ranged from 0.2 to 5.5 deaths per 100,000 person-years for males (far left of Fig 1a), and 0.1 to 2.2 deaths per 100,000 person-years for females (far left of Fig 1b). As of December 31, 2020, males and females in the 25 to 44-year age group, as well as females in their early-fifties, have experienced the highest mortality rates. Maximum mortality rates between 2003 and 2020 were 67.2 deaths per 100,000 person-years (95% credible interval: 55.3–81.5) among males around age 35 (far right of Fig 1a), and 16.8 deaths per 100,000 person-years (95% CI: 12.8–22.0) among females around age 37 (far right of Fig 1b) towards the end of 2020. In general, males experienced higher mortality rates than females over time and by age.

We also observed spikes in mortality in late-2017 to early-2019, as shown through dark patches in the heat maps of Fig 1a and 1b and corresponding with the increase of fentanyl exposure in Ontario. Subsequently, opioid-related mortality decreased in mid-2019 for both sexes to rates comparable to early-2018 for males and mid-2018 for females, but increased again in 2020 beyond rates previously observed.

**Fig 2:**
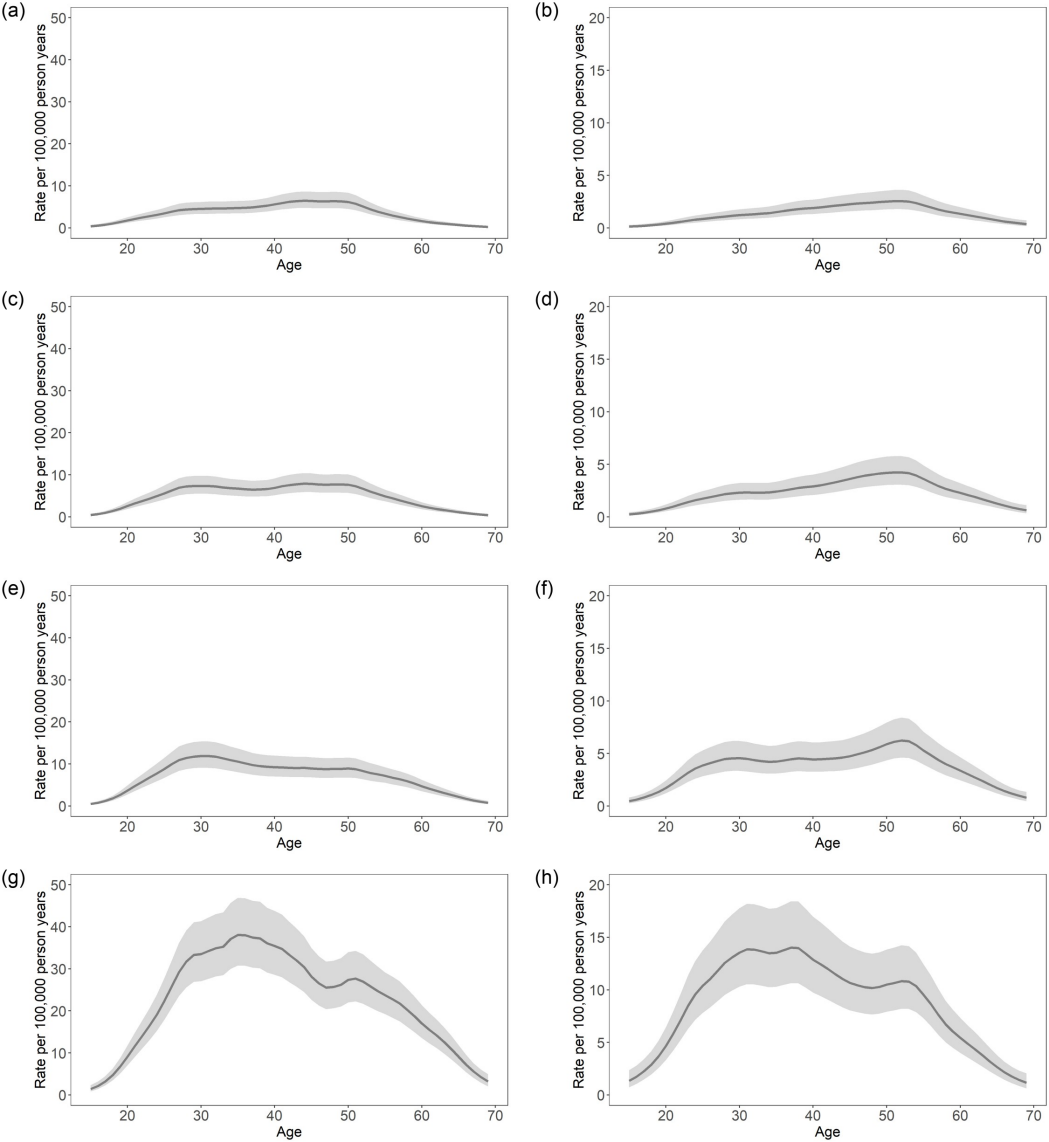
Opioid-related mortality rate per 100,000 person-years, by age for selected years (January 2005, 2010, 2015, 2020). (a) Males, January 2005. (b) Females, January 2005. (c) Males, January, 2010. (d) Females, January, 2010 (e) Males, January 2015. (f) Females, January 2015. (g) Males, January 2020. (h) Females, January 2020. Note: solid lines = median rate per 100,000 person-years; shaded regions = 95% CI.

The age distribution for mortality has shifted over time for both sexes. In order to better visualize these shifts, cross-sections of Fig 1a and 1b were plotted in Fig 2a–2h for January of selected years during the study period (2005, 2010, 2015, and 2020), with shaded CIs added for estimation uncertainties. In January 2005, the highest rates of opioid-related mortality were seen for mid-life individuals. Maximum rates reached 6.5 deaths per 100,000 person-years (95% CI: 4.8–8.7) among males around age 44 (the peak of Fig 2a) and 2.6 deaths per 100,000 person-years (95% CI: 1.8–3.6) among females around age 52 (the peak of Fig 2b). By January 2020, age distributions of opioid-related mortality became bimodal. For males, the maximum rate reached 38.0 deaths per 100,000 person-years (95% CI: 30.8–46.9) among individuals around age 35, and there was another peak that reached 27.7 deaths per 100,000 person-years (95% CI: 22.3–34.3) among individuals around age 51 (the two peaks of Fig 2g). Similarly, the distribution for females displayed peaks of 14.0 deaths and 10.8 deaths per 100,000 person-years among individuals around ages 37 and 52, respectively (95% CI 37 years: 10.6–18.4; 95% CI 52 years: 8.2–14.2) (the two peaks of Fig 2h). Tabulated rates from Fig 2a–2h can be found in S2 and S3 Tables.

**Fig 3:**
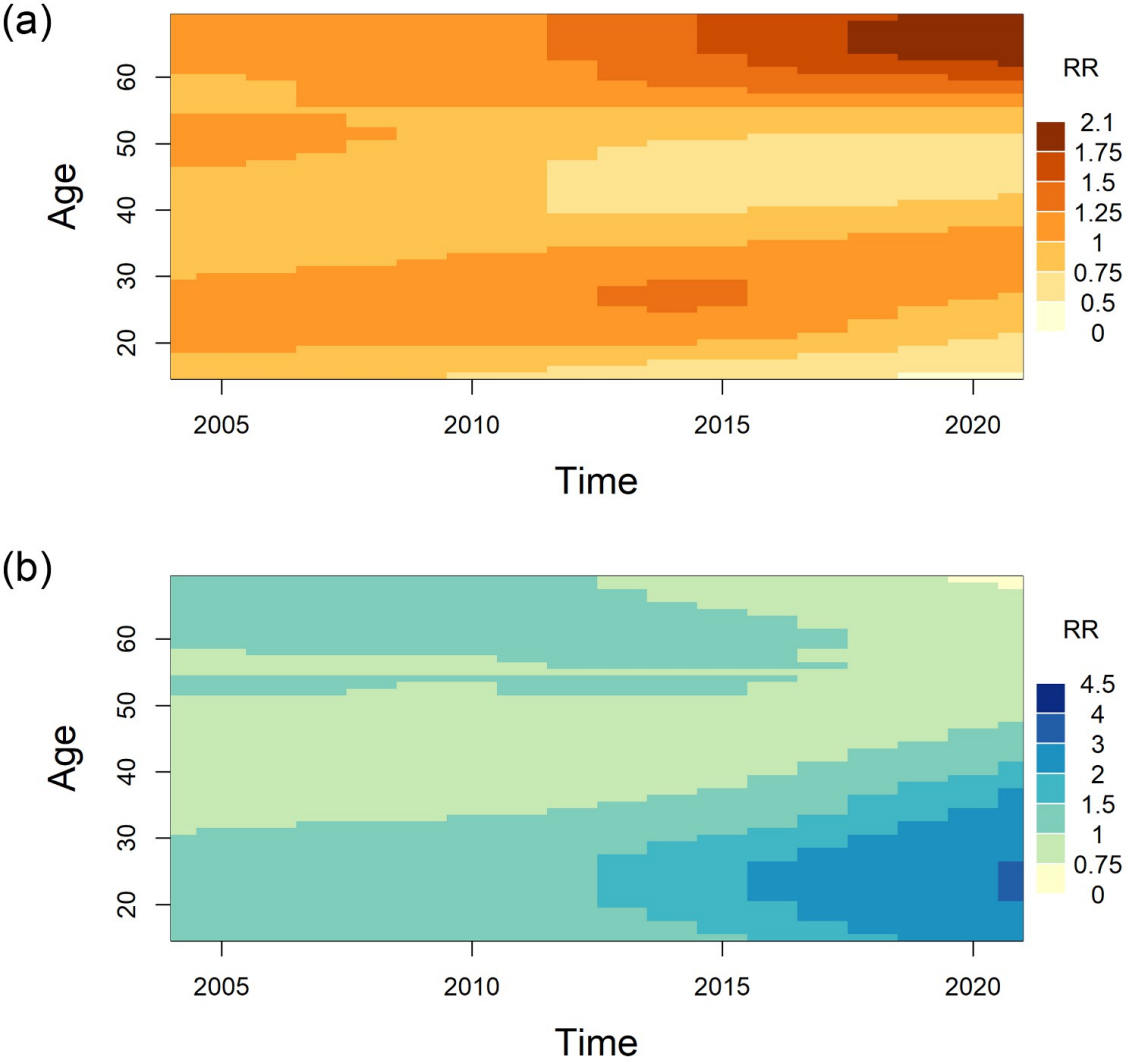
Interaction (two-dimensional random walk) between age and year of death (2003 to 2020; reference year = 2003, reference age = 55 years). (a) Males. (b) Females.

**Fig 4:**
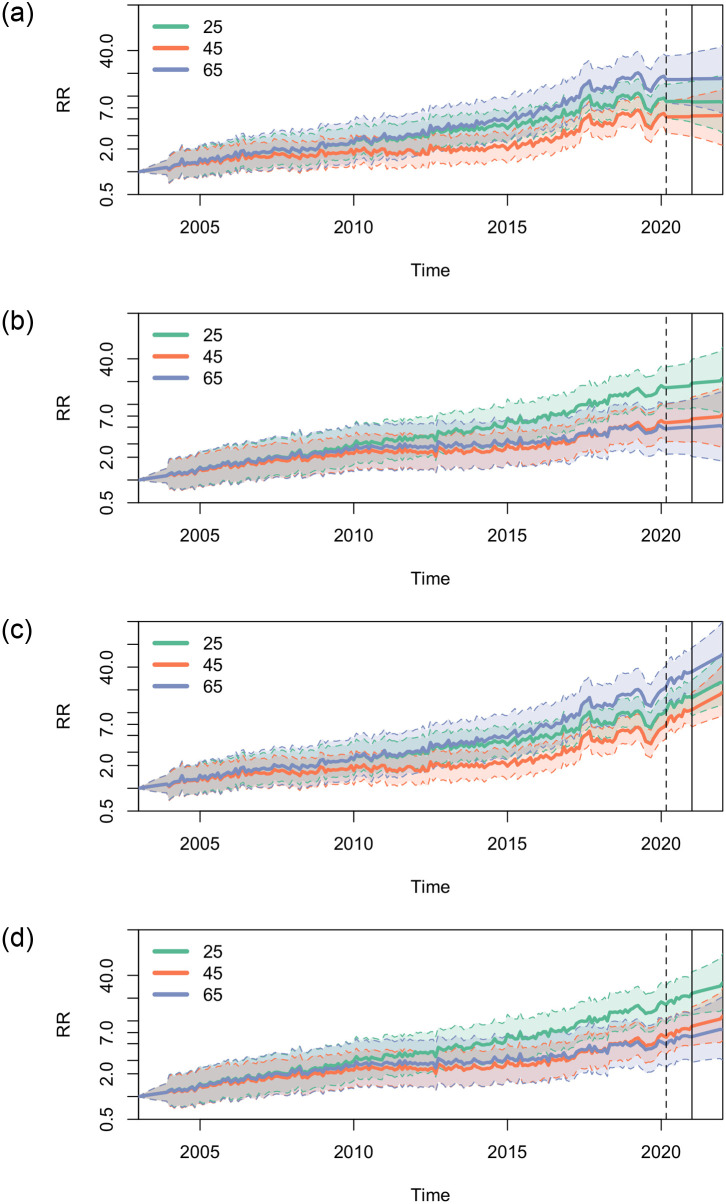
Estimates and exploration of relative rate of opioid-related mortality, by date of death in months for selected ages (January 2003 to December 2022; reference month = January 2003, reference age = 55 years) using pre- and post-pandemic data. (a) Males, pre-pandemic data. (b) Females, pre-pandemic data. (c) Males, post-pandemic data. (d) Females, post-pandemic data. Note: solid age lines = median relative rate; shaded regions = 95% CI; dotted vertical line = February 29, 2020; solid vertical line = December 31, 2020.

To understand how temporal changes in mortality differ by age, in Fig 3a and 3b we created heat maps of the interaction between age and year of death (corresponding to the term *exp*[*W*_*i*_(*a*, *k*)] in the S1 Appendix) with age 55 and 2003 as the reference for each sex. The largest increases in opioid-related mortality were among males in the 20 to 35-year age group and males older than 55 years (dark patches in Fig 3a), while for females, increases in mortality were concentrated among females younger than 35 years (dark patches in Fig 3b). In Fig 4c and 4d, we additionally plotted the relative mortality rates over time without main effects for age (corresponding to the term *exp*[*V*_*i*_(*t*) + *W*_*i*_(*a*, *k*) + *Z*_*it*_ − *Z*_*i*1_] the S1 Appendix) for selected ages (25, 45, and 65 years) in comparison to January 2003 for each sex. As of December 2020, the maximum relative rate of the three ages plotted was 33.8 (95% CI: 17.7–64.5) among males aged 65 compared with males aged 55 in January 2003 (up to the solid vertical line in Fig 4c). Conversely, females aged 25 had the highest relative rate of 20.8 (95% CI: 11.6–37.5) by December 2020 compared with females aged 55 in January 2003 (up to the solid vertical line in Fig 4d).

### Exploration of short-term mortality trends

**Fig 5:**
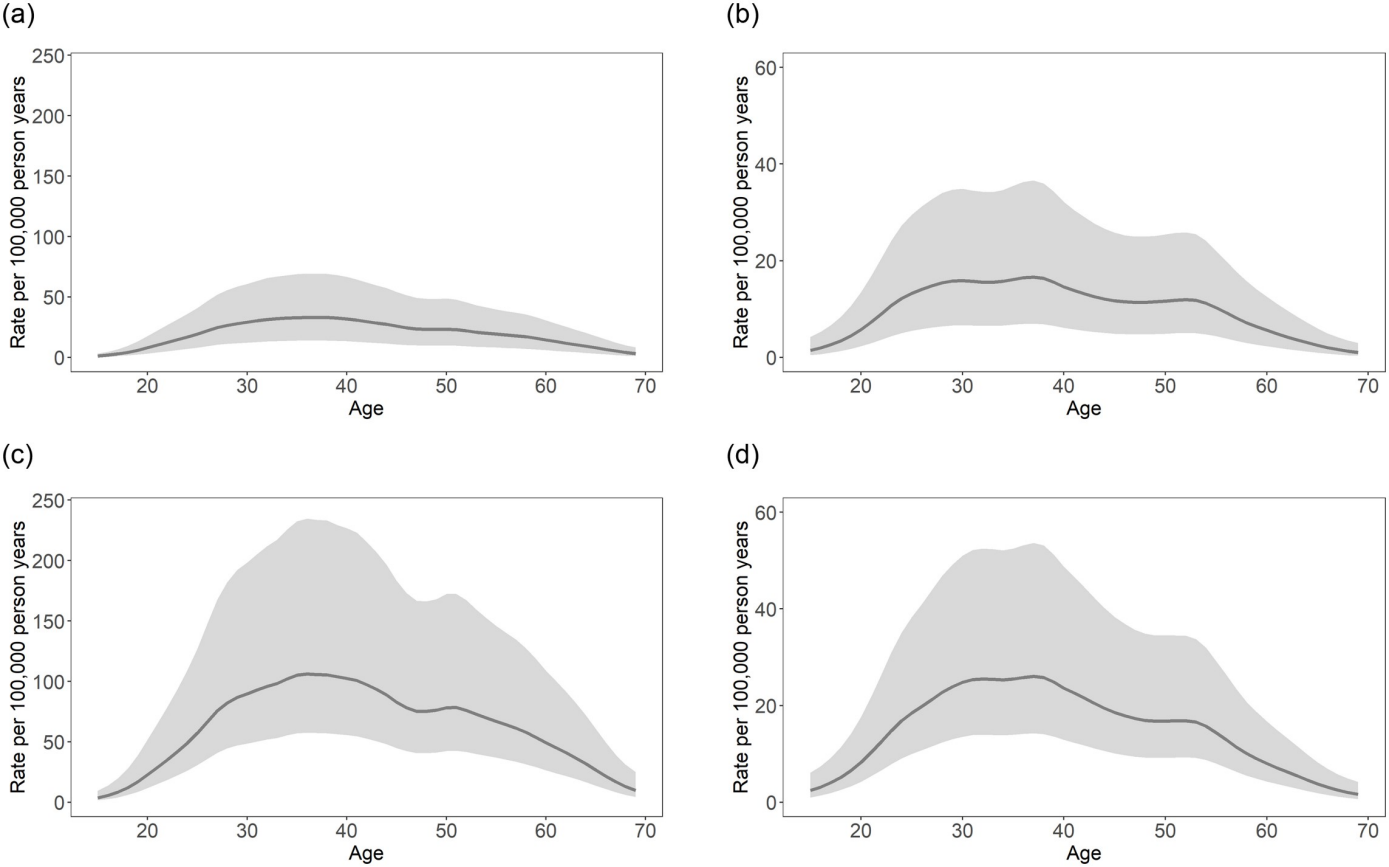
Exploration of opioid-related mortality rate per 100,000 person-years, by age for January 2022 using pre- and post-pandemic data. (a) Males, January 2022, pre-pandemic data. (b) Females, January 2022, pre-pandemic data. (c) Males, January 2022, post-pandemic data. (d) Females, January 2022, post-pandemic data. Note: solid lines = median rate per 100,000 person-years; shaded regions = 95% CI.

Based on the observed data and resulting parameters from the models, our models estimate that opioid-related mortality among the younger population will continue to grow. Specifically, age distributions of mortality rates are expected to become less bimodal, and instead peak among individuals in the 25 to 44-year age group, assuming trends continue without disturbance. This was observed for both sexes, as well as both excluding (Fig 5a and 5b) and including (Fig 5c and 5d) post-pandemic data from March 2020 to December 2020 in the model estimations. Further, comparing the maximum rates in Fig 5a and 5c, as well as Fig 5b and 5d, current trends could lead to mortality rates that are more than quadruple those of pre-pandemic (January 2003 to February 2020) estimations for males, and almost double those of pre-pandemic estimations for females, respectively, by the end of 2022.

From the models we also estimate based on pre-pandemic data that relative rates for both males and females might have remained relatively stable over the next 2 years (after the solid vertical line in Fig 4a and 4b). However the width of the CIs also encompass the possibility of increases or decreases in rates. Including post-pandemic data in the model estimation, males and females of all ages are estimated to experience increases in mortality over the next 2 years (after the solid vertical line in Fig 4c and 4d).

## Discussion

The application of a Bayesian model with random walks allowed us to graphically produce smooth surfaces for mortality over age and time, giving a more intuitive understanding of how the distribution has changed over the past two decades. Despite the fact that deaths did not occur at each single age and time point, mortality rates were estimated by borrowing information from adjacent ages and time through implementation of two-dimensional random walks on a grid made up of all combinations of age and time. This allowed for flexible curves showing the interaction effect and how the distribution of mortality changed over time. In particular, a shift in the age distribution of opioid-related mortality has occurred in Ontario. There has been a trend towards greater mortality among individuals in the 25 to 44-year age group compared to 18 years ago, when the highest mortality rates were among individuals in the 45 to 54-year age group. We observed decreases in mortality in mid-2019, however mortality rates increased again in 2020 during the COVID-19 pandemic.

Peer-reviewed studies that have examined trends in opioid-related mortality over time have mostly been conducted in the United States [11–13, 16, 19, 20, 23, 24, 27], with few in Canada [21], Australia [22], and New Zealand [14]. Additionally, several reports on opioid-related mortality have been published at the provincial and national levels in Canada [5, 6, 25, 28, 29]. Similar to our results, the studies that examined opioid-related mortality by sex consistently found absolute death rates to be higher for males compared to females [11, 14, 16, 20, 22, 23]. Several studies also found trends of shifting age distributions of opioid-related mortality over time. Olfson et al. [12] and Gomes et al. [20] examined mortality in the United States for the periods 2000–2017 and 2001–2016, respectively. Both studies found that by the end of the study period, the 25 to 34-year age group had surpassed all other age groups in mortality. Gomes et al. also conducted an equivalent analysis of opioid-related mortality in Ontario for the period 2000–2015, finding the same result [21]. Conversely, studies by Roxburgh et al. from Australia [22] and Shipton et al. from New Zealand [14] concluded that mortality rates remained highest among individuals in the 35 to 54-year age group. However, both studies only considered data up to 2012; in our analysis the shift in age distribution appeared to occur around 2011 for males and 2018 for females. This change in demographic is particularly worrisome alongside estimates that the proportion of deaths involving opioids in the 24 to 35-year age group in Ontario has increased from 1 in 8 deaths in 2010 to 1 in 6 deaths in 2015 [21]. In the United States this proportion is even higher, with 1 in 5 deaths among 24 to 35 year olds involving opioids [20], consequently leading to high rates of years of life lost from premature mortality [20, 21].

Regarding relative increases in opioid-related mortality, Gomes et al. found that in Ontario, individuals in the 15 to 24-year and 55 to 64-year age groups experienced the largest relative increases over time [21], while in the United States, the largest relative increases were observed for individuals in the 55 to 64-year and 65-year or older age groups [20]. Other studies from the United States have similarly seen larger relative increases among older adults compared to young adults [12, 16, 19, 23, 24, 27], and these findings are hypothesized to be evidence of an aging population that is increasingly being prescribed opioids for pain and other comorbidities [27, 30]. However further considering sex, we observed that while older males had the greatest relative increases in mortality over time compared to younger and middle-aged males, younger females had the greatest relative increases compared to older and middle-aged females. This may be the result of younger females facing different challenges than older females such as pregnancy/parenting, greater job loss, increased risk of mental health issues, and stigma around accessing supports for substance use treatment and harm reduction, which all may contribute to larger increases in opioid-related mortality and may also be further exacerbated by the pandemic [6, 31–34].

The long-term shift in the age distribution of opioid-related mortality towards younger individuals likely corresponds to changes in regional prescribing practices and most common substances in the unregulated drug supply. Prescription opioid-related mortality tends to be highest among older adults [11, 13, 16, 19, 22, 23, 27], whereas unregulated opioid-related mortality (e.g., fentanyl) tends to be highest among younger adults [11, 13, 16, 22, 27]. The U.S. has observed an overall decline in prescribing rates with substantial variation between states [9]; consequently, variation in trends of opioid-related mortality has been seen across the country [13, 16, 35]. Within Canada, we have similarly seen differences in prescribing practices between provinces. For example, the rate of opioids dispensed in British Columbia has been lower than that of Ontario [36]. Accordingly, trends in prescription opioid-related deaths have been more stable in British Columbia compared to Ontario [15]. Of note, we observed a more persistent bimodal distribution over time for females, where the shift towards greater opioid-related mortality among younger females did not occur until around seven years after the shift for males. This observation may be due to females being more likely to be prescribed opioids [31], and thus older females experiencing continued prescription opioid-related mortality over a longer period compared to older males [22].

This study has several strengths, including the use of a novel Bayesian modelling approach to assess trends by age, sex, and time, as well as to explore trends into the future. The use of random walks not only allowed us to include age and time as continuous predictors in the model in order to provide a granular picture of how mortality distributions have changed smoothly over time, but also made the model more flexible to motivate signals from the data. Few studies have examined age and sex trends simultaneously [13, 16], and we did not identify any studies that modeled trends by age, sex, and continuous time together. Additionally, we did not need to rely on ICD codes to identify opioid-related deaths, as the Office of the Chief Coroner for Ontario conducts thorough investigations of all non-natural deaths. Furthermore, as we had near-complete mortality data up to December 2020, we were also able to consider pre-COVID-19 and post-COVID-19 courses of mortality trends, which is of growing public health interest during the ongoing pandemic. We identified only one other analysis that modelled the impact of COVID-19 on mortality trends, with explorations up to the end of 2021 [28].

The study also contains some limitations that must be acknowledged. First, we were unable to add the effects of other predictors in our model, such as opioid type, socioeconomic status, ethnicity or comorbidities, due to data availability and the complexity of the modelling approach. The marginalized study population further limits the ability to link neighbourhood-level indicators derived for postal codes across Ontario (e.g., income), as mortality records from the cohort are more likely to have missing or inaccurate residential information compared to mortality records from the general population. Data availability also rendered us unable to examine simultaneous trends in opioid use disorder by age, sex and time in Ontario, which may have provide further insights into the opioid-related mortality trends we observed [37, 38]. Second, our ability to estimate future trends was limited by current mortality data availability. We were only comfortable exploring trends 2 years into the future to 2022 (assuming trends will continue without disturbance), after which credible intervals became too wide to be informative. We also note that the model is not a mechanistic or mathematical model that aims to predict future mortality with confidence, in which case the source and composition of drugs in the unregulated drug market would be significant contributors. Third, our model assumed that the variance of the random walks remained constant over time, however, the stochastic process of mortality rates before the pandemic may differ from the stochastic process of mortality rates during the pandemic. Relaxing this assumption might allow the model to better capture patterns of mortality, although more data would be required during the pandemic period to estimate the variances separately.

## Conclusion

This analysis may inform a refocusing of public health strategy to address the opioid overdose crisis—programs and policies for reducing opioid-related mortality should take into consideration its changing age distribution. Targeting individuals in the 25 to 44-year age group is of greater importance now than was the case previously, thus strategies should be developed to effectively reach younger adults with health and social supports such as harm reduction measures and treatment for opioid use disorder. Older males in the 55 to 69-year age group experienced relative increases in opioid-related mortality rates greater than that of their younger counterparts, thus they also represent an important cohort and may require alternate strategies tailored to their age group.

## Supporting information

S1 Fig Estimates of relative rates of opioid-related mortality by calendar month (reference = average of 12 months). (a) Males. (b) Females.
(ZIP)

S2 Fig Priors and posterior distributions for the standard deviations of random effects. (a) random walk on age. (b) iid random effect for time. (c) random walk on time. (d) two-dimensional random walk for age-time interaction. Note: red dashed lines = priors; black solid lines = posterior distributions of standard deviations.
(ZIP)

S3 Fig Fitted mortality for males aged 35 (line) overlaid with raw mortality data for males aged 33 to 37 with time binned at 3-month intervals (points). (TIFF)

S1 Table Ontario population estimates by age group for males and females in 2003 and 2020. (DOCX)

S2 Table Male opioid-related mortality rates per 100,000 person-years, by age for selected years (January 2005, 2010, 2015, 2020). (DOCX)

S3 Table Female opioid-related mortality rates per 100,000 person-years, by age for selected years (January 2005, 2010, 2015, 2020). (DOCX)

S1 Appendix (DOCX)
