# Supplementary figures and images for: Identifying the changing age distribution of opioid-related mortality with high-frequency data
Source: PLoS One. 2022 Apr 20;17(4):e0265509. doi: 10.1371/journal.pone.0265509 (PMC9020746; doi:10.1371/journal.pone.0265509)

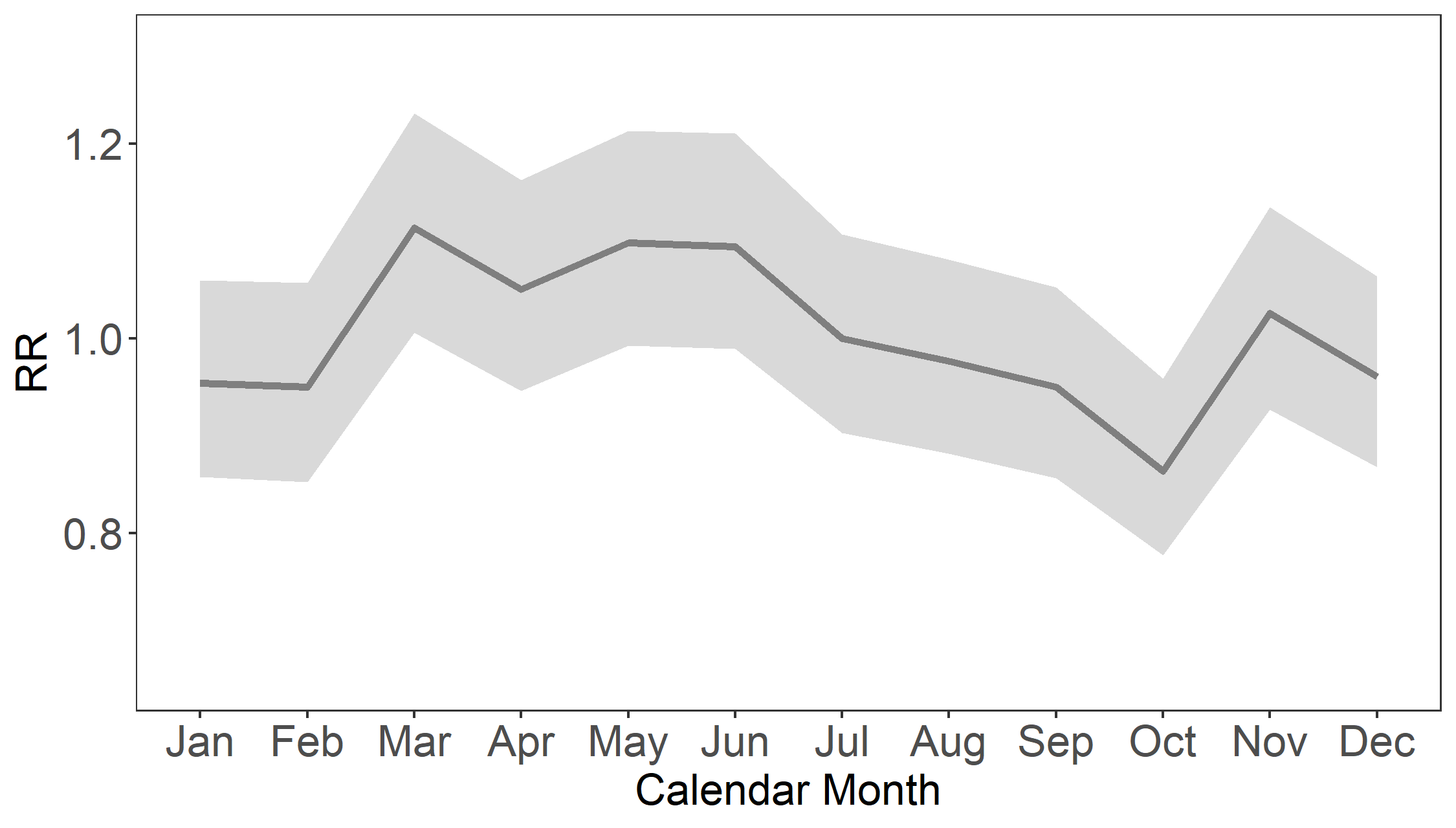

Supplement: S1 Fig — (a) Males. (b) Females. (ZIP) [file pone.0265509.s001.zip › S1a_Fig.tiff]

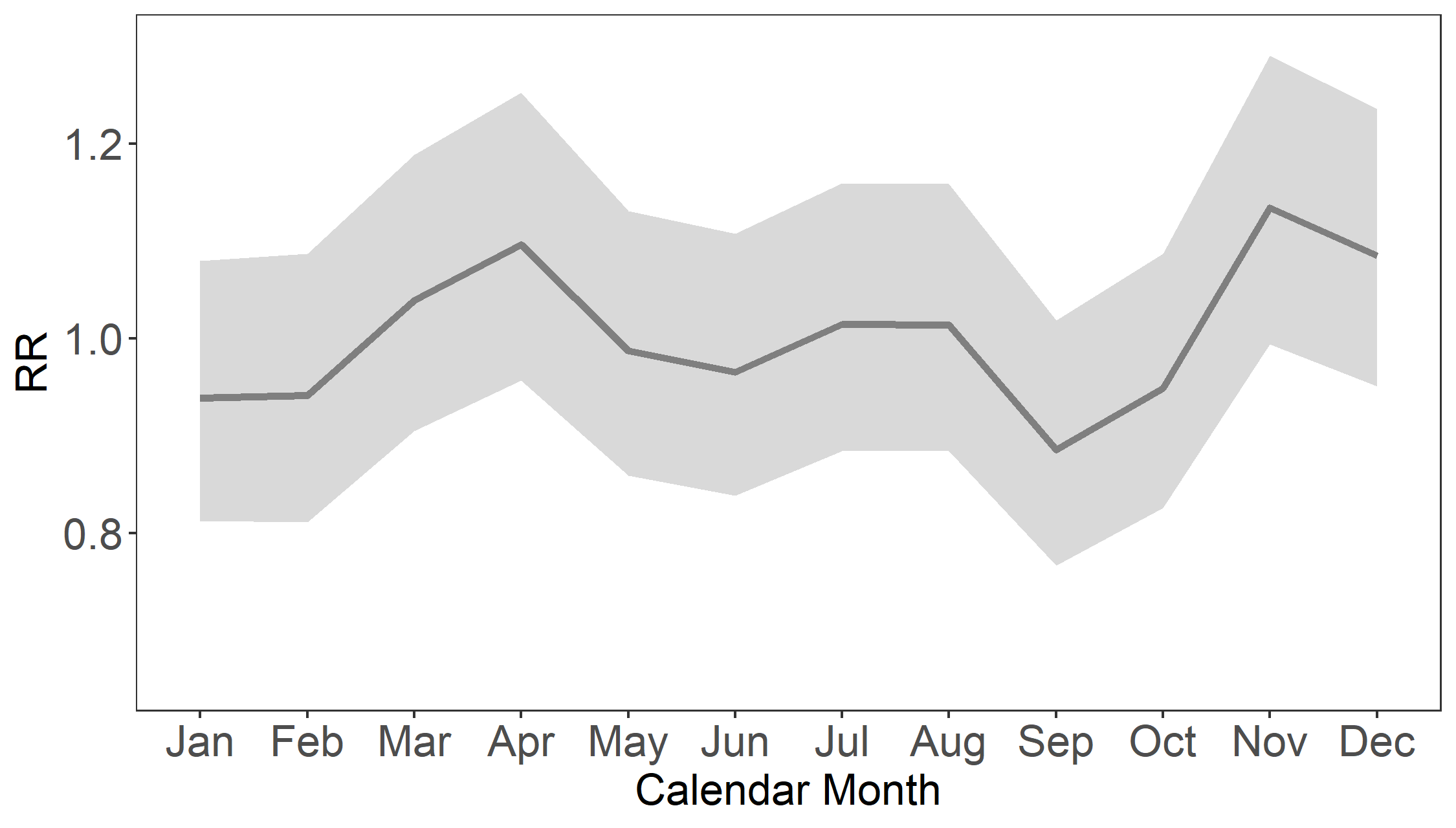

Supplement: S1 Fig — (a) Males. (b) Females. (ZIP) [file pone.0265509.s001.zip › S1b_Fig.tiff]

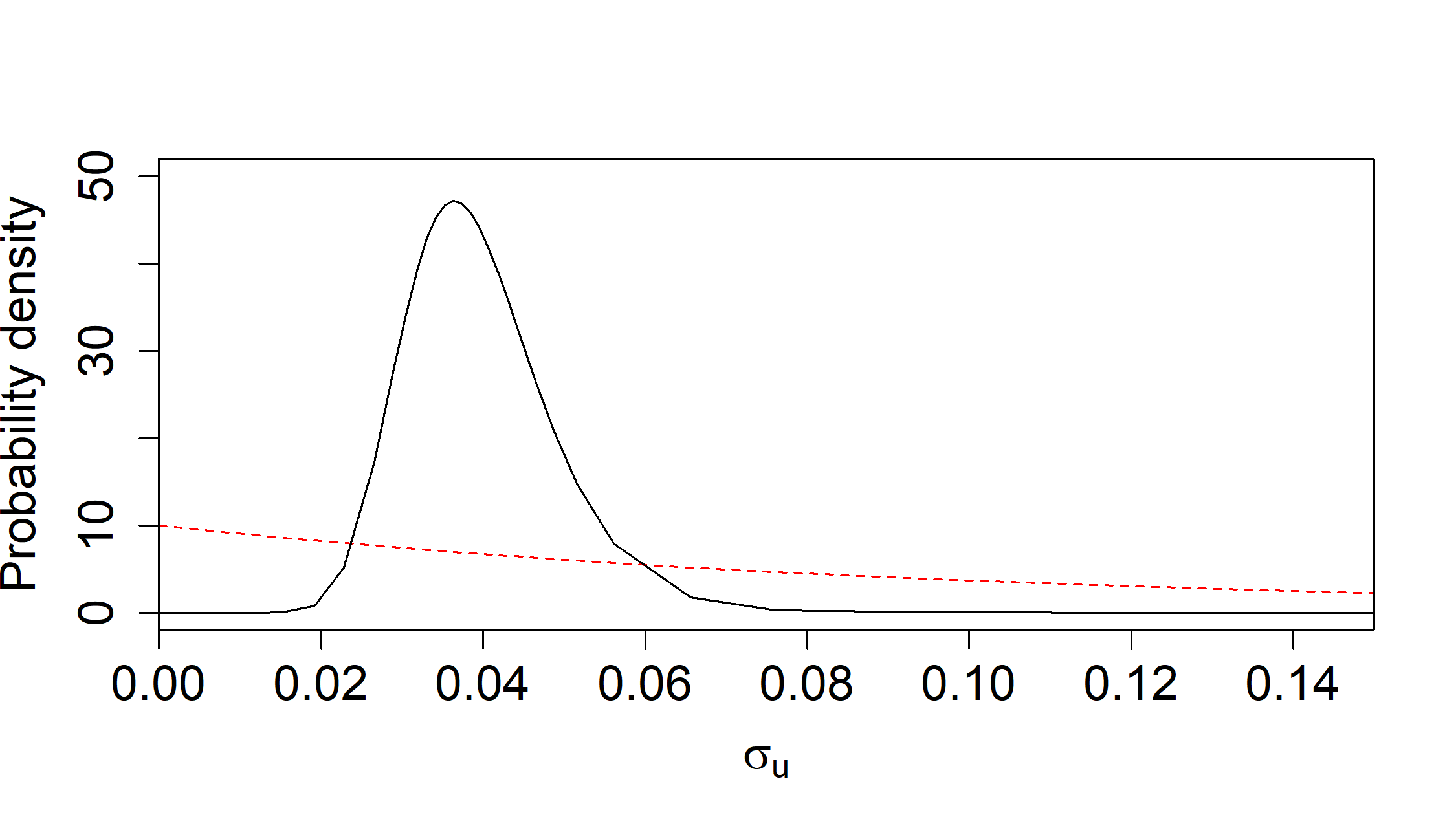

Supplement: S2 Fig — (a) random walk on age. (b) iid random effect for time. (c) random walk on time. (d) two-dimensional random walk for age-time interaction. Note: red dashed lines = priors; black solid lines = posterior distributions of standard deviations. (ZIP) [file pone.0265509.s002.zip › S2a_Fig.tiff]

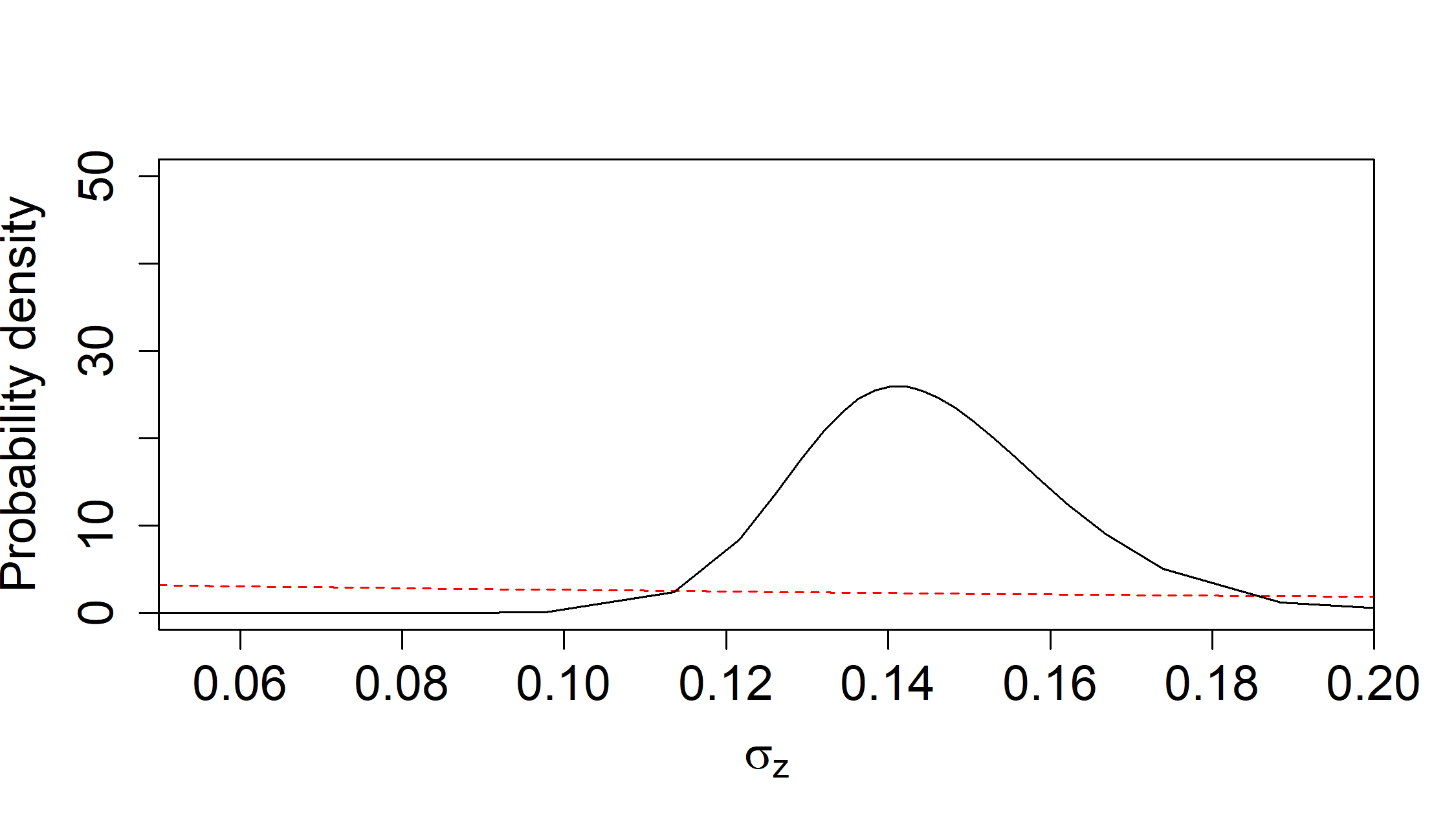

Supplement: S2 Fig — (a) random walk on age. (b) iid random effect for time. (c) random walk on time. (d) two-dimensional random walk for age-time interaction. Note: red dashed lines = priors; black solid lines = posterior distributions of standard deviations. (ZIP) [file pone.0265509.s002.zip › S2b_Fig.tiff]

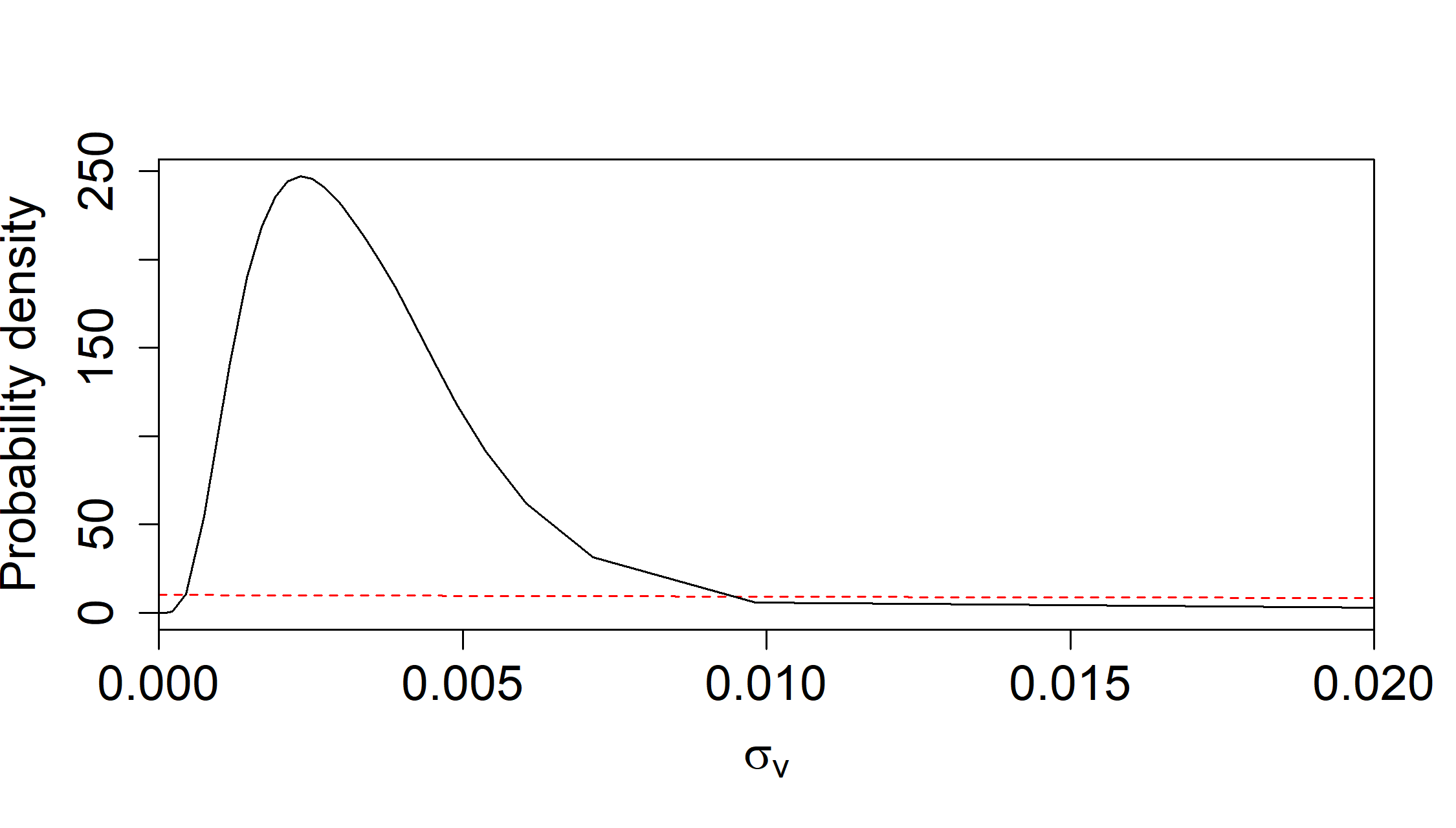

Supplement: S2 Fig — (a) random walk on age. (b) iid random effect for time. (c) random walk on time. (d) two-dimensional random walk for age-time interaction. Note: red dashed lines = priors; black solid lines = posterior distributions of standard deviations. (ZIP) [file pone.0265509.s002.zip › S2c_Fig.tiff]

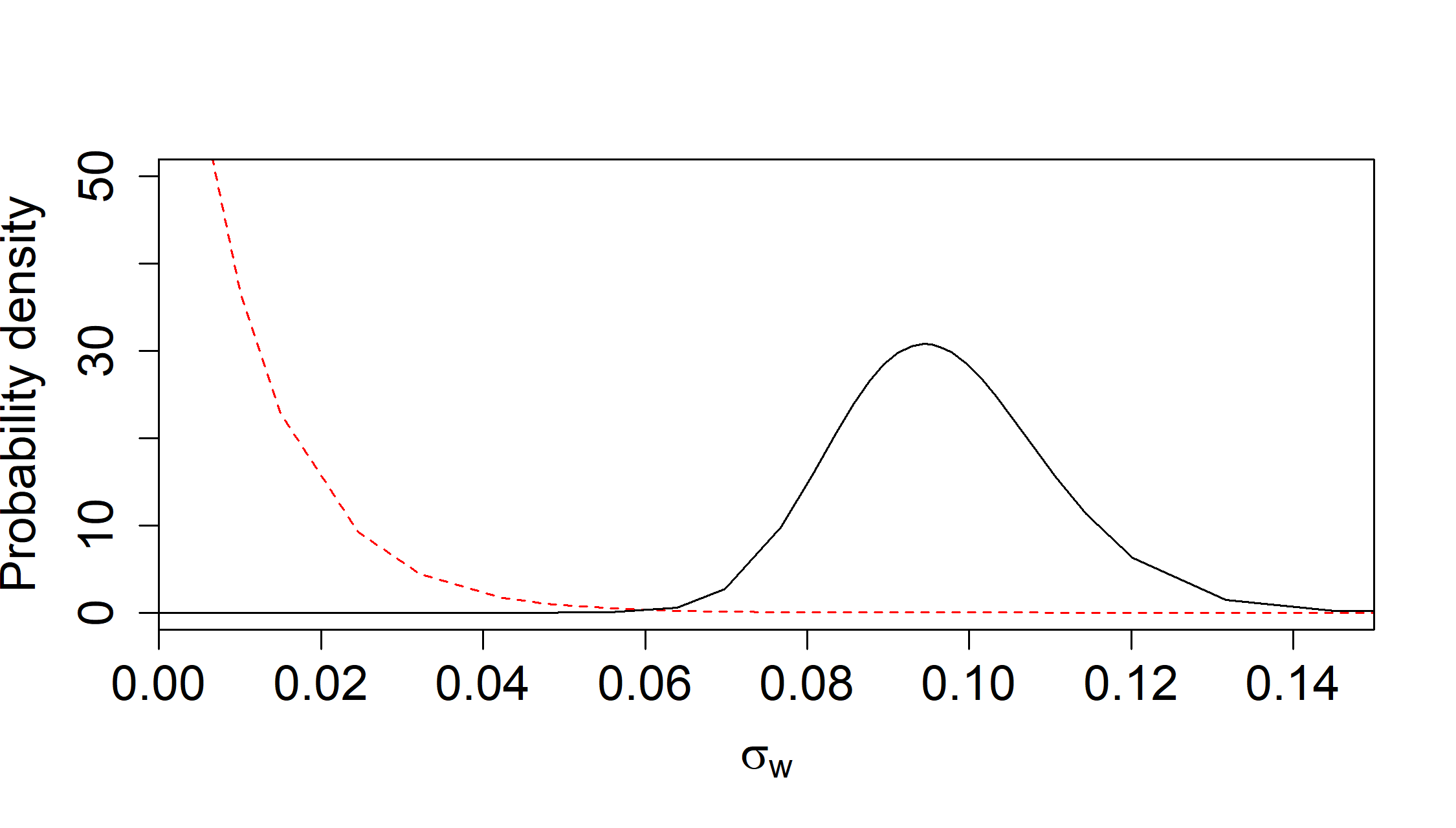

Supplement: S2 Fig — (a) random walk on age. (b) iid random effect for time. (c) random walk on time. (d) two-dimensional random walk for age-time interaction. Note: red dashed lines = priors; black solid lines = posterior distributions of standard deviations. (ZIP) [file pone.0265509.s002.zip › S2d_Fig.tiff]

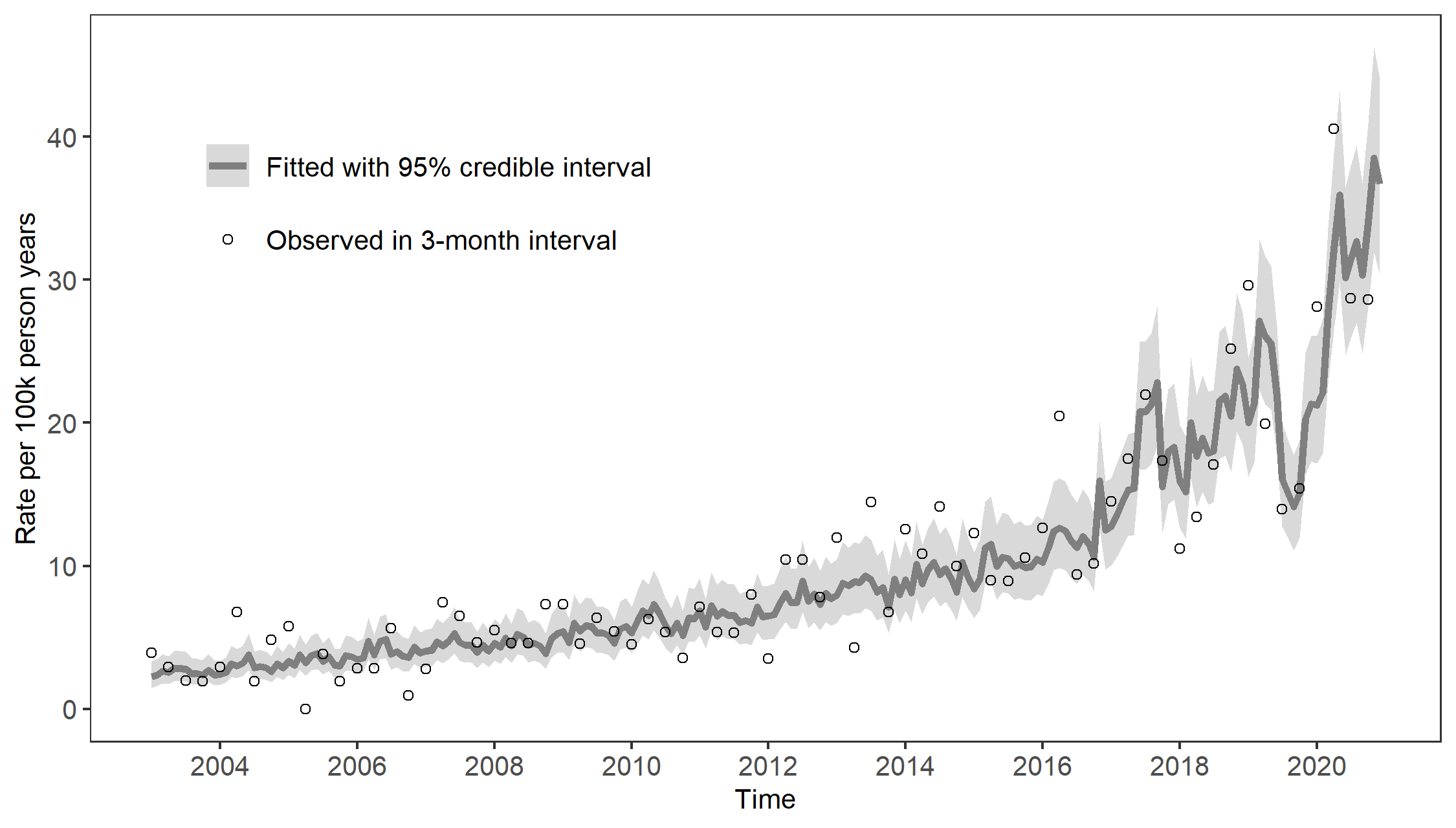

Supplement: S3 Fig — (TIFF) [file pone.0265509.s003.tiff]
