# Supplementary material for: Identifying the changing age distribution of opioid-related mortality with high-frequency data
Source: PLoS One. 2022 Apr 20;17(4):e0265509. doi: 10.1371/journal.pone.0265509 (PMC9020746; doi:10.1371/journal.pone.0265509)
Supplement: S1 Table — (DOCX) [file pone.0265509.s004.docx]

**S1 Table. Ontario population estimates by age group for males and females in 2003 and 2020**

| **Age group** | **Males** | | **Females** | |
| --- | --- | --- | --- | --- |
|  | **2003** | **2020** | **2003** | **2020** |
| 15 – 24 years | 855,665 | 943,945 | 815,974 | 883,039 |
| 25 – 34 years | 865,962 | 1,059,014 | 859,683 | 1,069,800 |
| 35 – 44 years | 1,050,726 | 957,507 | 1,038,833 | 1,000,992 |
| 45 – 54 years | 877,550 | 951,671 | 896,075 | 979,744 |
| 55 – 64 years | 602,866 | 1,029,756 | 623,980 | 1,056,984 |
| 65 – 69 years | 212,447 | 396,514 | 232,236 | 429,651 |
