# Supplementary material for: Identifying the changing age distribution of opioid-related mortality with high-frequency data
Source: PLoS One. 2022 Apr 20;17(4):e0265509. doi: 10.1371/journal.pone.0265509 (PMC9020746; doi:10.1371/journal.pone.0265509)
Supplement: S2 Table — (DOCX) [file pone.0265509.s005.docx]

**S2 Table. Male opioid-related mortality rates per 100,000 person-years, by age for selected years (January 2005, 2010, 2015, 2020)**

| **Age** | **2005** | | **2010** | | **2015** | | **2020** | |
| --- | --- | --- | --- | --- | --- | --- | --- | --- |
|  | **Rate** | **95% CI** | **Rate** | **95% CI** | **Rate** | **95% CI** | **Rate** | **95% CI** |
| 15 years | 0.4 | 0.2 – 0.7 | 0.4 | 0.3 – 0.7 | 0.5 | 0.3 – 0.8 | 1.4 | 0.8 – 2.3 |
| 16 years | 0.6 | 0.3 – 0.9 | 0.7 | 0.4 – 1.0 | 0.8 | 0.5 – 1.1 | 2.1 | 1.4 – 3.2 |
| 17 years | 0.8 | 0.5 – 1.2 | 1.0 | 0.7 – 1.4 | 1.2 | 0.8 – 1.7 | 3.2 | 2.3 – 4.5 |
| 18 years | 1.1 | 0.7 – 1.6 | 1.4 | 1.0 – 2.0 | 1.8 | 1.3 – 2.5 | 4.7 | 3.5 – 6.3 |
| 19 years | 1.4 | 1.0 – 2.0 | 1.9 | 1.4 – 2.7 | 2.7 | 2.0 – 3.6 | 6.7 | 5.1 – 8.8 |
| 20 years | 1.8 | 1.3 – 2.6 | 2.6 | 1.9 – 3.5 | 3.7 | 2.8 – 4.9 | 9.1 | 7.0 – 11.7 |
| 21 years | 2.2 | 1.5 – 3.0 | 3.2 | 2.3 – 4.3 | 4.7 | 3.6 – 6.3 | 11.6 | 9.0 – 14.7 |
| 22 years | 2.5 | 1.8 – 3.5 | 3.8 | 2.8 – 5.1 | 5.8 | 4.4 – 7.6 | 14.0 | 11.1 – 17.7 |
| 23 years | 2.8 | 2.0 – 3.9 | 4.4 | 3.2 – 5.8 | 6.8 | 5.1 – 8.8 | 16.5 | 13.1 – 20.7 |
| 24 years | 3.1 | 2.2 – 4.3 | 4.9 | 3.7 – 6.6 | 7.7 | 5.9 – 10.1 | 19.1 | 15.3 – 23.9 |
| 25 years | 3.5 | 2.5 – 4.8 | 5.6 | 4.1 – 7.4 | 8.8 | 6.7 – 11.4 | 22.3 | 17.8 – 27.7 |
| 26 years | 3.9 | 2.8 – 5.3 | 6.3 | 4.7 – 8.3 | 9.9 | 7.6 – 12.9 | 25.8 | 20.7 – 31.9 |
| 27 years | 4.2 | 3.1 – 5.8 | 6.9 | 5.2 – 9.2 | 10.9 | 8.4 – 14.2 | 29.2 | 23.6 – 36.1 |
| 28 years | 4.4 | 3.2 – 6.0 | 7.2 | 5.4 – 9.6 | 11.4 | 8.8 – 14.8 | 31.7 | 25.6 – 39.1 |
| 29 years | 4.5 | 3.3 – 6.1 | 7.4 | 5.5 – 9.8 | 11.8 | 9.0 – 15.2 | 33.3 | 26.9 – 41.0 |
| 30 years | 4.5 | 3.3 – 6.2 | 7.4 | 5.5 – 9.8 | 11.9 | 9.1 – 15.4 | 33.5 | 27.1 – 41.3 |
| 31 years | 4.6 | 3.4 – 6.3 | 7.3 | 5.5 – 9.7 | 11.9 | 9.1 – 15.4 | 34.2 | 27.6 – 42.2 |
| 32 years | 4.6 | 3.4 – 6.3 | 7.2 | 5.4 – 9.6 | 11.7 | 9.0 – 15.2 | 34.9 | 28.2 – 43.0 |
| 33 years | 4.6 | 3.4 – 6.3 | 7.0 | 5.2 – 9.3 | 11.3 | 8.7 – 14.7 | 35.2 | 28.5 – 43.4 |
| 34 years | 4.7 | 3.5 – 6.4 | 6.8 | 5.1 – 9.0 | 10.9 | 8.3 – 14.1 | 37.1 | 30.0 – 45.7 |
| 35 years | 4.7 | 3.5 – 6.4 | 6.7 | 5.0 – 8.9 | 10.6 | 8.1 – 13.7 | 38.0 | 30.8 – 46.9 |
| 36 years | 4.8 | 3.5 – 6.5 | 6.6 | 4.9 – 8.7 | 10.2 | 7.8 – 13.2 | 38.0 | 30.7 – 46.8 |
| 37 years | 4.9 | 3.6 – 6.6 | 6.5 | 4.8 – 8.6 | 9.7 | 7.4 – 12.6 | 37.5 | 30.3 – 46.2 |
| 38 years | 5.1 | 3.7 – 6.9 | 6.5 | 4.9 – 8.7 | 9.5 | 7.2 – 12.3 | 37.2 | 30.1 – 46.0 |
| 39 years | 5.3 | 3.9 – 7.2 | 6.6 | 5.0 – 8.8 | 9.3 | 7.1 – 12.1 | 36.1 | 29.1 – 44.5 |
| 40 years | 5.6 | 4.2 – 7.6 | 6.9 | 5.2 – 9.1 | 9.2 | 7.0 – 12.0 | 35.5 | 28.6 – 43.8 |
| 41 years | 5.9 | 4.4 – 8.0 | 7.3 | 5.5 – 9.6 | 9.2 | 7.0 – 11.9 | 34.7 | 28.0 – 42.9 |
| 42 years | 6.2 | 4.6 – 8.3 | 7.6 | 5.7 – 10.0 | 9.1 | 6.9 – 11.8 | 33.3 | 26.8 – 41.2 |
| 43 years | 6.4 | 4.7 – 8.6 | 7.8 | 5.8 – 10.3 | 9.0 | 6.9 – 11.7 | 32.0 | 25.7 – 39.6 |
| 44 years | 6.5 | 4.8 – 8.7 | 7.9 | 5.9 – 10.4 | 9.1 | 6.9 – 11.8 | 30.4 | 24.5 – 37.7 |
| 45 years | 6.4 | 4.8 – 8.6 | 7.8 | 5.9 – 10.3 | 8.9 | 6.8 – 11.6 | 28.2 | 22.6 – 34.9 |
| 46 years | 6.3 | 4.7 – 8.5 | 7.7 | 5.8 – 10.2 | 8.8 | 6.7 – 11.5 | 26.6 | 21.3 – 33.0 |
| 47 years | 6.3 | 4.7 – 8.5 | 7.6 | 5.7 – 10.1 | 8.8 | 6.7 – 11.4 | 25.5 | 20.5 – 31.7 |
| 48 years | 6.4 | 4.7 – 8.6 | 7.7 | 5.8 – 10.2 | 8.8 | 6.7 – 11.4 | 25.7 | 20.6 – 31.9 |
| 49 years | 6.3 | 4.7 – 8.5 | 7.7 | 5.8 – 10.1 | 8.8 | 6.7 – 11.4 | 26.2 | 21.1 – 32.5 |
| 50 years | 6.2 | 4.5 – 8.3 | 7.6 | 5.7 – 10.1 | 8.9 | 6.8 – 11.5 | 27.4 | 22.0 – 34.0 |
| 51 years | 5.7 | 4.2 – 7.8 | 7.3 | 5.5 – 9.6 | 8.8 | 6.7 – 11.4 | 27.7 | 22.3 – 34.3 |
| 52 years | 5.1 | 3.7 – 6.9 | 6.7 | 5.0 – 8.8 | 8.3 | 6.4 – 10.8 | 27.0 | 21.7 – 33.4 |
| 53 years | 4.4 | 3.2 – 6.0 | 6.0 | 4.5 – 8.0 | 7.9 | 6.0 – 10.2 | 25.8 | 20.8 – 32.0 |
| 54 years | 3.9 | 2.8 – 5.3 | 5.4 | 4.1 – 7.2 | 7.5 | 5.8 – 9.7 | 24.7 | 20.0 – 30.5 |
| 55 years | 3.4 | 2.5 – 4.6 | 4.9 | 3.7 – 6.5 | 7.2 | 5.6 – 9.3 | 23.8 | 19.3 – 29.2 |
| 56 years | 3.0 | 2.1 – 4.1 | 4.4 | 3.3 – 5.9 | 6.8 | 5.2 – 8.8 | 22.8 | 18.4 – 28.1 |
| 57 years | 2.6 | 1.8 – 3.6 | 3.9 | 2.9 – 5.3 | 6.4 | 4.8 – 8.3 | 21.8 | 17.5 – 27.0 |
| 58 years | 2.2 | 1.6 – 3.1 | 3.5 | 2.5 – 4.7 | 5.9 | 4.5 – 7.7 | 20.3 | 16.3 – 25.3 |
| 59 years | 1.9 | 1.3 – 2.7 | 3.0 | 2.2 – 4.1 | 5.3 | 4.0 – 7.0 | 18.8 | 15.0 – 23.4 |
| 60 years | 1.6 | 1.1 – 2.3 | 2.5 | 1.8 – 3.5 | 4.7 | 3.5 – 6.2 | 17.0 | 13.5 – 21.3 |
| 61 years | 1.4 | 0.9 – 2.0 | 2.2 | 1.6 – 3.0 | 4.1 | 3.1 – 5.5 | 15.5 | 12.3 – 19.6 |
| 62 years | 1.2 | 0.8 – 1.7 | 1.9 | 1.3 – 2.6 | 3.6 | 2.7 – 4.8 | 14.2 | 11.2 – 18.0 |
| 63 years | 1.0 | 0.7 – 1.5 | 1.6 | 1.1 – 2.2 | 3.1 | 2.3 – 4.2 | 12.6 | 9.9 – 16.1 |
| 64 years | 0.8 | 0.5 – 1.3 | 1.4 | 0.9 – 1.9 | 2.6 | 1.9 – 3.6 | 10.9 | 8.5 – 14.1 |
| 65 years | 0.7 | 0.4 – 1.1 | 1.1 | 0.8 – 1.6 | 2.2 | 1.6 – 3.0 | 9.1 | 7.0 – 11.8 |
| 66 years | 0.5 | 0.3 – 0.9 | 0.9 | 0.6 – 1.3 | 1.7 | 1.2 – 2.4 | 7.3 | 5.5 – 9.6 |
| 67 years | 0.4 | 0.2 – 0.7 | 0.7 | 0.4 – 1.1 | 1.3 | 0.9 – 1.9 | 5.6 | 4.1 – 7.6 |
| 68 years | 0.3 | 0.2 – 0.6 | 0.5 | 0.3 – 0.9 | 1.0 | 0.7 – 1.5 | 4.3 | 2.9 – 6.0 |
| 69 years | 0.3 | 0.1 – 0.5 | 0.4 | 0.2 – 0.7 | 0.8 | 0.5 – 1.2 | 3.2 | 2.0 – 5.0 |
