# Supplementary material for: Identifying the changing age distribution of opioid-related mortality with high-frequency data
Source: PLoS One. 2022 Apr 20;17(4):e0265509. doi: 10.1371/journal.pone.0265509 (PMC9020746; doi:10.1371/journal.pone.0265509)
Supplement: S1 Appendix — (DOCX) [file pone.0265509.s007.docx]

S1 Technical Appendix

# Model & Notation

Let $Y_{i}(a,t)$ and $P_{i}(a,t)$ be the mortality count and person-years offset (derived from population data), respectively, for age group $a$ in month $t$ (since January 2003) and sex $i$. The data includes opioid-related deaths between ages 15 and 69 from January 2003 to December 2020. Giving $a\in\left\{ 15, 16,\ldots, 69 \right\}, i\in\left\{ M, F \right\}, t\in\left\{ 1, 2, 3,\ldots, 216 \right\} ($where $t=1$ for January 2003)$, k\in\left\{ 2003, 2004,\ldots, 2020 \right\}$, and writing $\lambda_{i}(a,t)$ as the corresponding monthly mortality rate, the model is:

$$\begin{matrix} Y_{i}(a,t)\sim& \text{Poisson}\left[ \lambda_{i}(a,t)P_{i}(a,t) \right] \\ \text{log}\left[ \lambda_{i}(a,t) \right]= & X_{t}\beta_{i}+U_{i}(a)+V_{i}(t)+W_{i}(a,k)+Z_{it} \\ U_{i}(\cdot)\sim& \text{RW2}(\sigma_{U}^{2}) \\ V_{i}(\cdot)\sim& \text{RW2}(\sigma_{V}^{2}) \\ W_{i}(\cdot,\cdot)\sim& \text{RW2D}(\sigma_{W}^{2}) \\ Z_{it}\sim& \text{iid N}(0,\sigma_{Z}^{2}). \end{matrix}$$

- $X_{t}$ is a vector of covariates with an indicator variable for each calendar month.
- The $\beta_{i}$ are the regression coefficients for $X_{t}$, which differ by sex $i$.
- The notation RW2 refers to a second-order random walk (or random slope) model with, for example:

$$U_{i}(a)-2U_{i}(a-1)+U_{i}(a-2)\sim\text{N}(0,\sigma_{U}^{2})$$

- RW2D is a two-dimensional (but first-order) random walk to model interaction between age and time in years:

$$W_{i}(a,k)|W_{i}(u,v);u,v\neq a,k\sim\text{N}(\text{mean}\{W_{i}(a-1,k),W_{i}(a+1,k),W_{i}(a,k-1),W_{i}(a,k+1)\},\sigma_{W}^{2})$$

- $Z_{it}$ is a normally distributed random effect to capture the random fluctuations over time.

Note that there are effectively two separate models for males and females with a common set of variance parameters for the random effects.

# Constraints

The following constrains are applied to aid model identifiability and interpretation:

- The intercept is set to 0.
- The indicator variables for calendar month are constrained to sum to zero.
- For random walk on age, the age effect is set to 0 at age 55.
  - $U(55)=0$
- For random walk on time, the time effect is set to 0 in January 2003.
  - $V(0)=0$
- For two-dimensional random walks on a regular grid (interaction between age and time in years), the effect for year 2003 is set to 0 for each age, and the effect for age 55 is set to 0 for each year.
  - $W(55,k)=0$
  - $W(a,0)=0$

# Priors & Inference

A non-informative prior is used for the fixed effects $\beta_{i}$. The standard deviations of the random effects have moderately informative exponential priors, which encourage the standard deviations to be close to zero and the random effects to be roughly constant. More specifically, the priors are:

$$\begin{matrix} \beta& \sim N(0,{10}^{5}) \\ \sigma_{Z} & \sim\text{Exp}\left[ \text{log}(1.2)\cdot\text{log}(2) \right] \\ \sigma_{U} & \sim\text{Exp}\left[ \text{log}(0.2)\cdot\text{log}(2) \right] \\ \sigma_{V} & \sim\text{Exp}\left[ \text{log}(0.2)\cdot\text{log}(2) \right] \\ \sigma_{W} & \sim\text{Exp}\left[ \text{log}(1.2)\cdot\text{log}(2) \right] \end{matrix}$$

The standard deviations have posterior medians $\text{log}(1.2)$ or $\text{log}(0.2)$; these values are multiplied by $\text{log}(2)$ to get the posterior means. The inferences are done via Integrated Nested Laplace Approximation (INLA) in R, which directly gives the posterior marginal distribution of the parameters instead of samples. Estimated mortality patterns are compared, plotted with credible intervals, and interpreted.
